# Detoxification and Immune Transcriptomic Response of the Gill Tissue of Bay Scallop (*Argopecten irradians*) Following Exposure to the Algicide Palmitoleic Acid

**DOI:** 10.3390/biom8040139

**Published:** 2018-11-06

**Authors:** Cheng Chi, Sib Sankar Giri, Jin Woo Jun, Hyoun Joong Kim, Sang Wha Kim, Jeong Woo Kang, Se Chang Park

**Affiliations:** 1 Laboratory of Aquatic Nutrition and Ecology, College of Animal Science and Technology, Nanjing Agricultural University, Weigang Road 1, Nanjing 210095, China; chicheng0421@126.com; 2 Laboratory of Aquatic Biomedicine, College of Veterinary Medicine and Research Institute for Veterinary Science, Seoul National University, Seoul 08826, Korea; giribiotek@gmail.com (S.S.G.); hjoong1@nate.com (H.J.K.); kasey.kim90@gmail.com (S.W.K.); kck90victory@naver.com (J.W.K.); 3 Department of Aquaculture, Korea National College of Agriculture and Fisheries, Jeonju 54874, Korea; advancewoo@hanmail.net

**Keywords:** algicide, palmitoleic acid, bay scallop, transcriptomic analysis

## Abstract

Palmitoleic acid (PA) is an effective algicide against *Alexandrium tamarense*. However, the toxicological mechanism of PA exposure is unclear. The transcript abundance and differentially expressed genes (DEGs) in gills of bay scallop were investigated following 80 mg/L PA exposure up to 48 h using the Illumina HiSeq 4000 deep-sequencing platform with the recommended read length of 100 bp. De novo assembly of paired-end reads yielded 62,099 unigenes; 5414 genes were identified as being significantly increased, and 4452 were decreased. Based on gene ontology classification and enrichment analysis, the ‘cellular process’, ‘metabolic process’, ‘response to stimulus’, and ‘catalytic process’ with particularly high functional enrichment were revealed. The DEGs, which are related to detoxification and immune responses, revealed that acid phosphatase, fibrinogen C domain-containing protein, cyclic AMP-responsive element-binding protein, *g*lutathione reductase, ATP-binding cassette, nuclear factor erythroid 2-related factor, NADPH2:quinone reductase, and cytochrome P450 4F22, 4B1, and 2C8-related gene expression decreased. In contrast, some genes related to glutathione *S*-transferase, C-type lectin, superoxide dismutase, toll-like receptors, and cytochrome P450 2C14, 2U1, 3A24 and 4A2 increased. The results of current research will be a valuable resource for the investigation of gene expression stimulated by PA, and will help understanding of the molecular mechanisms underlying the scallops’ response to PA exposure.

## 1. Introduction

In China, bivalves are among the most important commercial marine species, contributing a large proportion of the total shellfish production [1]. Furthermore, on account of the filter-feeding habits of bivalves, they are widely used for monitoring ocean pollution [2]. The scallop, as a crucial commercial marine species, is mainly distributed in coastal areas of Northeast Asia [3]. In addition to their economic value, scallops also act as model species in various toxicology studies [4,5] or as a sentinel organism in environmental monitoring programs [6]. In recent decades, the rapid expansion of industry and agriculture along with the population explosion has been accompanied by an apparent global increase in the occurrence, area, and detrimental effects of harmful algal blooms (HABs). Chemical methods of controlling HABs have been found, with toxic potential for aquatic species, posing environmental hazards. Therefore, in recent years, biological agents (especially bacteria or bacterial bioactive metabolites) are being proposed as alternative approaches for HABs outbreak control [7,8,9]. Previous studies have also reported that certain bacteria could inhibit or degrade HABs in the ocean [10,11]. *Alexandrium tamarense* is a harmful algal species in oceans which has led to substantial financial ruin in the aquaculture industry, as it can produce a paralytic shellfish toxin. Moreover, it can also cause human poisoning, and even death [12,13]. In recent years, palmitoleic acid (PA) was isolated and identified from *Vibrio* sp BS02 products as a bioactive compound against harmful algae. It was recommended to be applied for managing HABs in the China sea area [7]. At a concentration of 80 mg/L, PA could almost inhibit algal growth completely. Lysis of *A. tamarense* cells can be observed under light microscopy. In addition, PA can also effectively suppress other harmful algae growth (e.g., *A. minutum*, *Heterosigma akashiwo,* and *Asterionella japonica*) [7]. Nevertheless, PA exposure may cause secondary aquatic pollution. Thus, in order to investigate if and how it has affects other marine species, we studied the interaction between PA and scallop physiological responses, in which we measured various parameters, including the activities of acid phosphatase (ACP), alkaline phosphatase (ALP), superoxide dismutase (SOD), lysozymes, the levels of nitrite oxide (NO), lactate dehydrogenase (LDH), glutathione (GSH), and malondialdehyde (MDA) in our previous studies [13,14], which are related to immune, stress and detoxification responses. We accordingly found that PA could cause stress responses, had toxic effects, and even induced oxidative stress or metabolic disturbances of non-target marine species. However, how scallops respond to PA toxicity, and the details of their detoxification process during PA exposure, remain unclear, particularly the integral response at the transcriptional level. An understanding of the effects of PA exposure on the bay scallop is essential to establish effective measures to estimate its toxic potential. Therefore, in order to expand upon our previous work to further reveal potential impacts of PA exposure, we examined the transcriptomic responses of *Argopecten irradians* to PA exposure.

De novo sequencing offers an effective measure for obtaining full scallop transcriptome information. In this regard, platforms such as the Illumina HiSeq™ 4000 sequencing system facilitate extensive genome analysis at relatively low cost and high output [15]. Digital gene expression (DGE) analysis based on this sequencing platform, is applied for a growing number of aquatic organisms such as *Oryzias melastigma* [16], *Crassostrea gigas* [17], and *Chlamys farreri* [18] to investigate their responses to environmental stressors. The objective of this study was to develop a further understanding of the molecular responses of scallops upon exposure to PA. The previous studies found that 80 mg/L PA caused oxidative stress, stimulated the immune response, and was toxic to physiological function in *A. irradians* [13]. The gill acts as a defense barrier because its role in the filtration of suspended matter, and shows a high expression of putative immune-related genes [19]. Therefore, it was studied as the research target. Digital gene expression analysis was performed by using the Illumina HiSeq™ 4000 sequencing system, and then quantitative real-time PCR was conducted to verify several selected differentially expressed genes (DEGs). The objective of the current study was to assist future studies on the molecular mechanisms underlying the toxic effects of the algicide PA on bivalves, and investigating whether utilizing PA as an algicide poses a potential risk to marine production.

## 2. Results

### 2.1. Analysis of Differentially Expressed Genes Libraries

**Figure 1:**
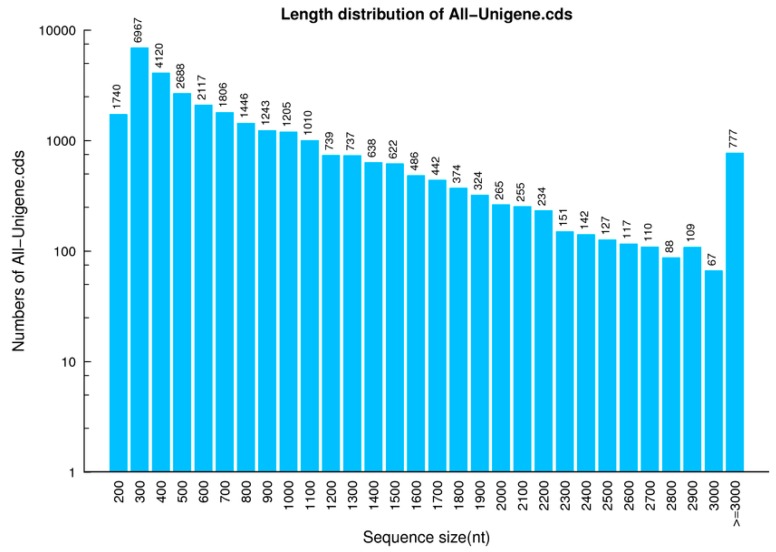
Unigene length distribution. The x-axis represents the length of unigenes. The *y*-axis represents the number of unigenes.

Two DGE libraries were constructed for the gills of control and PA-exposed scallops. After removing reads with adaptors, reads containing poly N, and low-quality reads from the raw data, a total of 9.16 Gb clean bases were generated. After filtering, reads quality metrics are shown as Table 1. Clean sequences from each library were assembled by the Trinity tool (version: v2.0.6), the assembly quality metrics are shown as Table 2. The two cDNA libraries were accordingly considered to be reliable. Finally, transcript sets from the two libraries were further merged to give 62,099 unigenes, which are shown in Table 3. The length distribution of unigene was shown in Figure 1.

### 2.2. Analysis of Sequences Mapping

**Figure 2:**
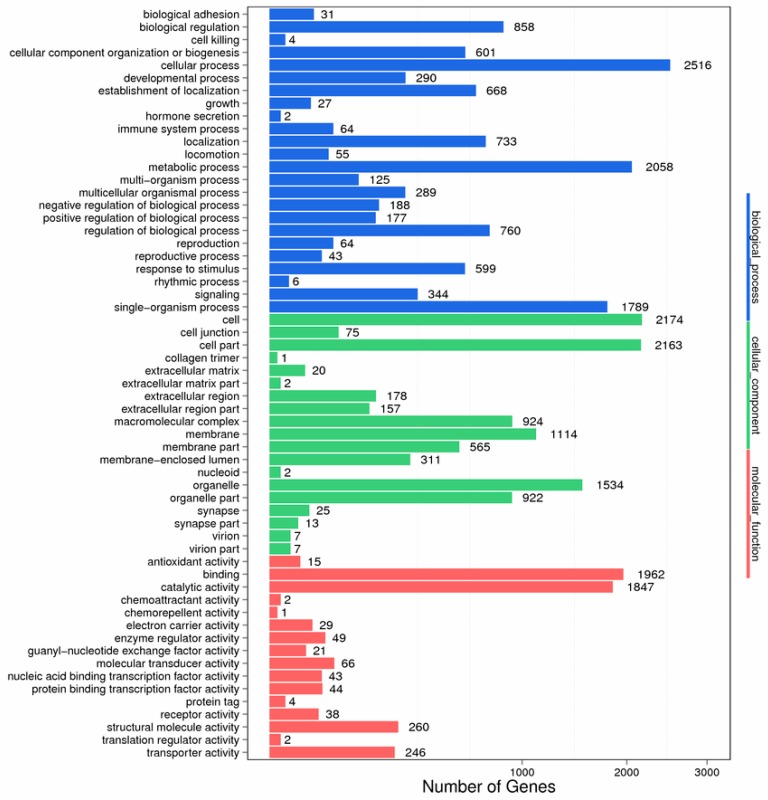
Gene Ontology (GO) annotation of all unigenes.

After assembly, functional annotation was performed by using the non-redundant protein sequence database (Nr), the NCBI nucleotide database (Nt), the Clusters of Orthologous Groups (COG), the Kyoto Encyclopedia of Genes and Genomes (KEGG), Gene Ontology (GO), Swissprot, and Interpro for unigenes. A total of 48.39% (30,049 unigenes) were annotated, of which 26,893 (43.31%) unigenes were annotated to the Nr; 10,803 (17.40%) to the Nt; 20,784 (33.47%) unigenes to Swiss-Prot; 20,274 (32.65%) unigenes to KEGG; 9420 (15.17%) unigenes to COG; 20,165 (32.47%) unigenes to Interpro; and 4199 (6.76%) unigenes to GO, respectively. For functional classification, 27,114 unigenes were totally annotated to the GO database. Among 59 functional groups, cellular process (2516 genes), metabolic process (2058), cell (2174), and catalytic activity (1962) showed considerable enrichment (Figure 2).

### 2.3. Differential Gene Expression Analysis

**Figure 3:**
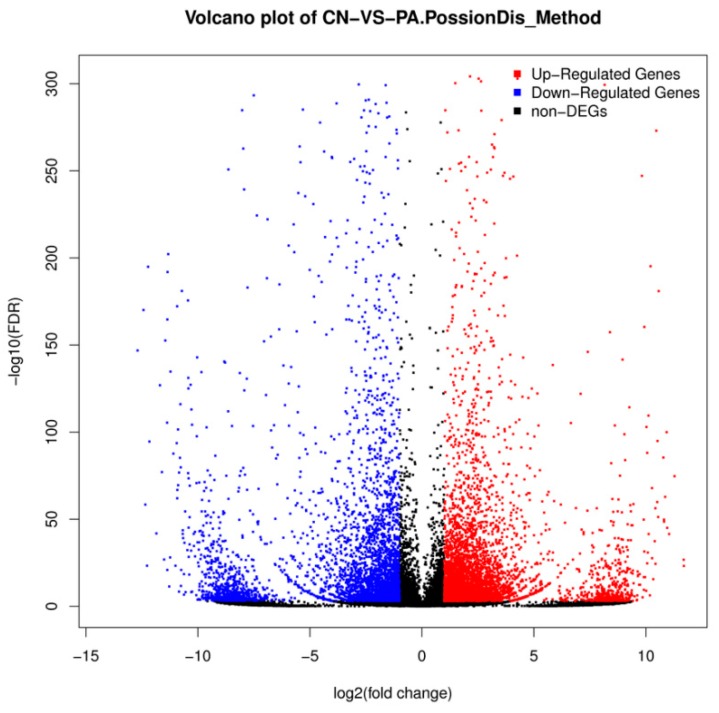
Volcano plot of differentially expressed genes (DEGs) between the control (CN) and PA-treated group. Blue, red and black points represent down regulated genes, up regulated genes and non-differential expression genes, respectively. FDR: false discovery rate.

**Figure 4:**
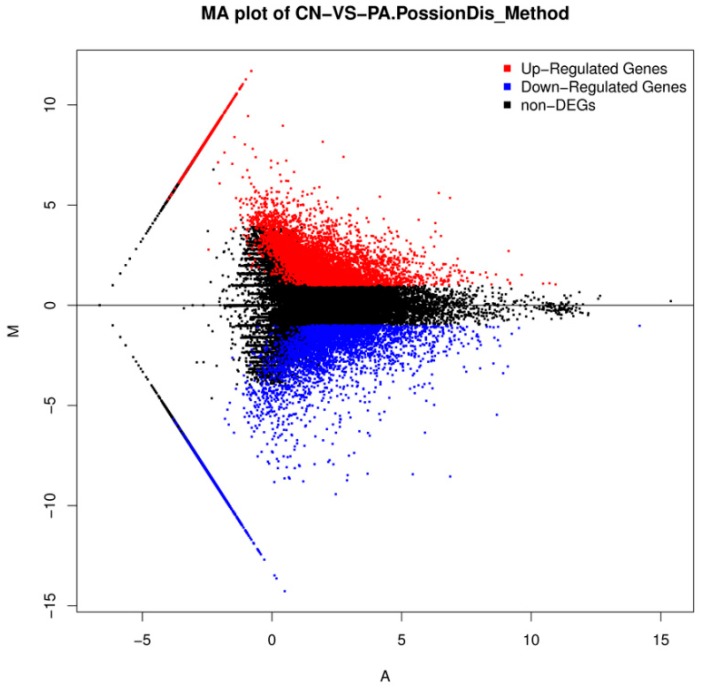
M (log ratio) and A (mean average) (MA) plot of DEGs between the control (CN) and the PA-treated group. The *x*-axis represents value A (log2 mean expression level). The y-axis represents value M (log2 transformed fold change). Blue, red, and black points represent down regulated genes, up regulated genes and non-differential expression genes, respectively.

The differential gene expression levels between two groups were calculated by using the Fragments Per Kilobase Million (FPKM) method (Figure 3 and Figure 4). A total of 9430 unigenes showed differential expression (fold changes larger than two and FDR ≤0.001) between the two groups. Among the unigenes, 5327 genes were found to be increased, while 4103 genes were found to be decreased (Supplementary file 1).

### 2.4. Enrichment and Pathway Analysis

**Figure 5:**
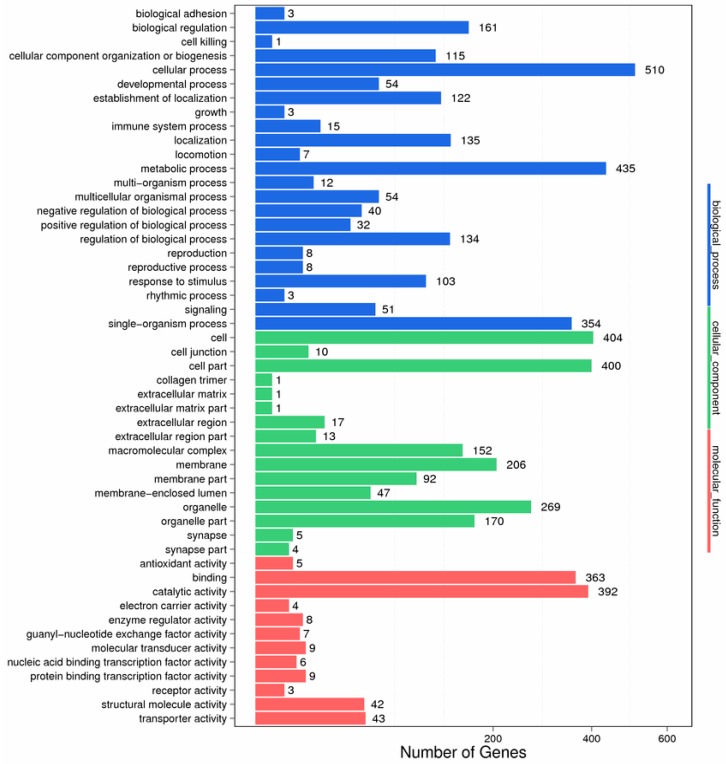
GO classification of the differentially expressed genes. The *x*-axis represents the GO term.

To investigate changes in the patterns of gene expression after PA exposure, all the DEGs to terms in the GO database were mapped. The considerable dominant GO terms in the gill after exposing to PA were involved to cellular process, metabolic process, single-organism process, cell, organelle, and catalytic activity (Figure 5).

**Figure 6:**
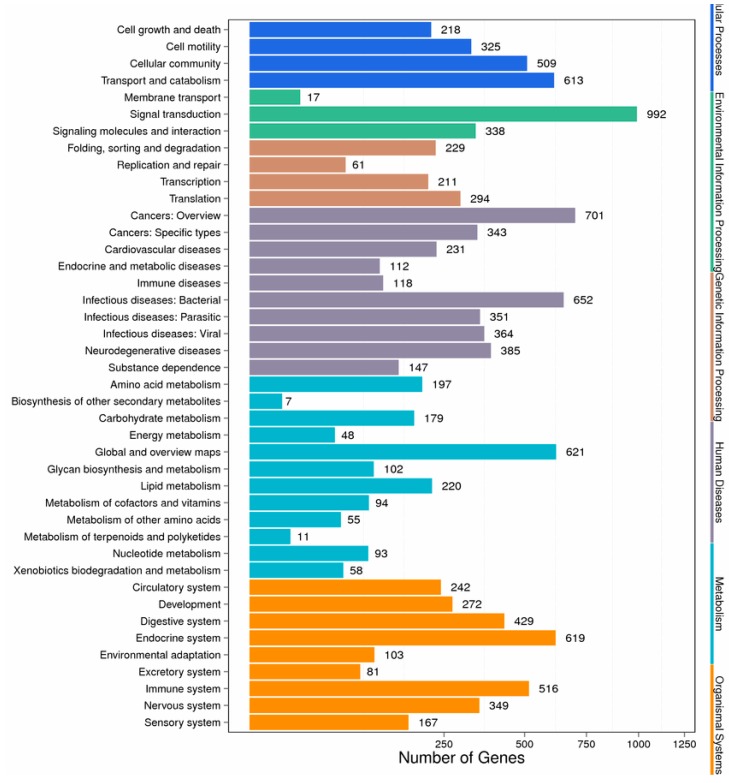
Pathway classification of differentially expressed genes. The *x*-axis shows the number of differentially expressed genes. The y-axis shows the pathway name.

**Figure 7:**
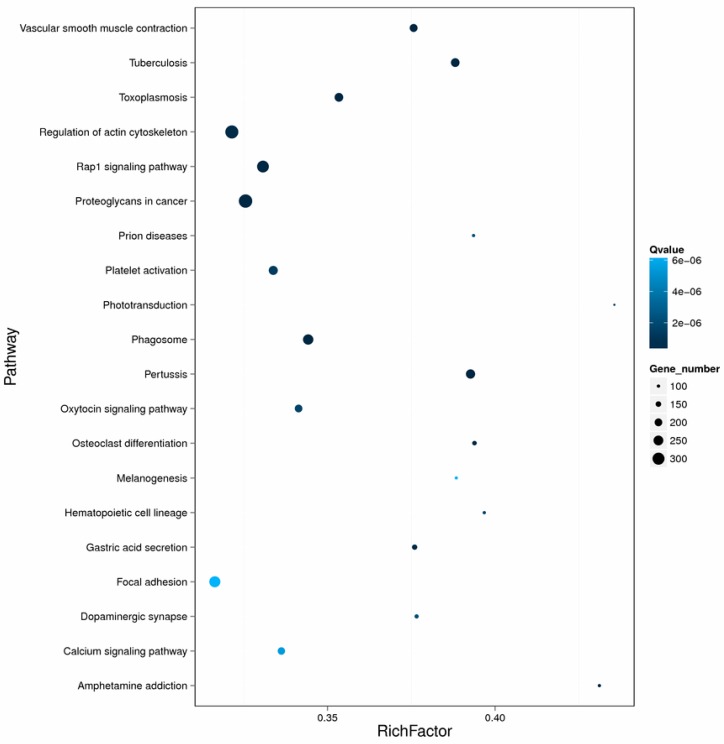
Enrichment of differentially expressed genes and pathways. The *x*-axis represents enrichment factor and the y-axis represents the pathway name. Coloring represents the Qvalue (high: white, low: blue), the lower Qvalue represents the more significant enrichment. The point size represents the differentially expressed genes number (more: big, less: small).

In order to identify the functional categories of the gene expression changes associated with PA exposure, unigenes were assigned to various metabolic pathways based on the KEGG database. Among all the DEGs, 4953 genes were assigned to 304 pathways in the KEGG database. One hundred and twenty-one metabolic pathways were significantly enriched (corrected *p*-value <0.05). The pathway classification and functional enrichment results are shown in Figure 6 and Figure 7. The expression of detoxification and immune-related genes including acid phosphatase, fibrinogen C domain-containing protein, cyclic AMP-responsive element-binding protein 3, glutathione reductase, ATP-binding cassette, nuclear factor erythroid 2-related factor, NADPH2:quinone reductase, and cytochrome P450 4F22, 4B1, and 2C8 decreased following PA exposure. On the contrary, some genes related to toll-like receptors, glutathione *S*-transferase, superoxide dismutase, C-type lectin, and cytochrome P450 2C14, 2U1, 3A24, and 4A2 were induced (Table 4).

### 2.5. Identification of Genes Related to Palmitoleic Acid-Induced Stress Response

**Figure 8:**
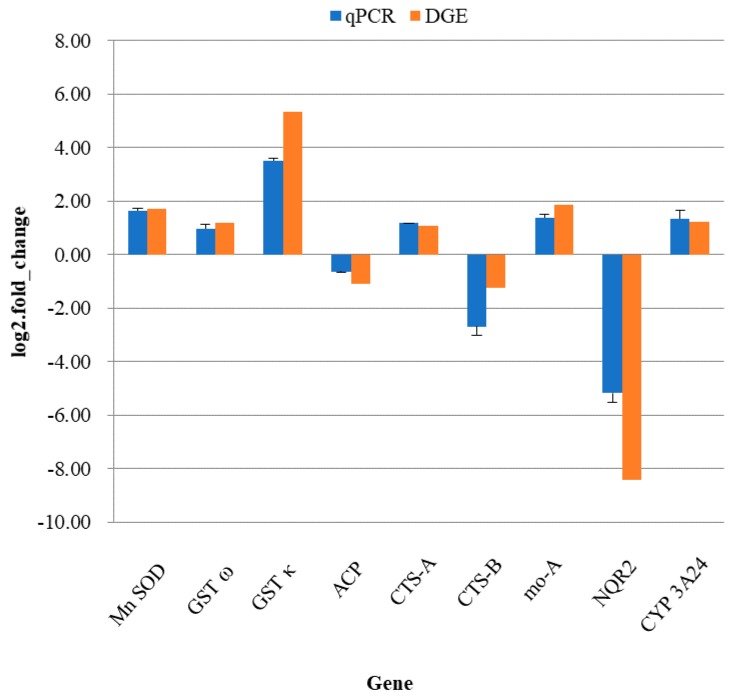
Results of quantitative real time PCR validation. The *y*-axis represents the gene expressed log2 (fold change) and the *x*-axis is the gene name. Mn SOD = manganese superoxide dismutase, GST ω = glutathione-*S*-transferase ω, GST κ= glutathione-*S*-transferase κ, ACP = acid phosphatase, CTS-A = cathepsin A, CTS-B = cathepsin B, mo-A = monoamine oxidase A, NQR2 = NADPH2: quinone reductase, CYP3A24 = cytochrome P450 3A24.

The relative expression level of nine genes (five increased and four decreased genes) from the two DGE libraries were assessed by qPCR. The melting-curve analysis of qPCR revealed a single product for all genes. Fold changes from qPCR were compared with the DGE analysis results. As shown in Figure 8, the data revealed that *Mn SOD*, *GST κ*, *GST ω*, *CTS-A*, *mo-A*, and *CYP3A24* were significantly up-regulated, while *ACP*, *CTS-B*, and *NQR2* were down-regulated. The genes exhibited a concordant direction in both the DGE library and qPCR analysis, and the correlation coefficient between DGE and qPCR results was 0.96 (*p* < 0.001).

## 3. Discussion

The following four main steps: hazard identification, dose–response assessment, exposure assessment, and risk characterization are widely acknowledged to be a standard method and entails of environmental risk assessment. Moreover, a variety of pollutants were identified to cause immunotoxic effects on species [20]. To confront the negative effects of HABs on humans or aquaculture industry, biological control measures are supposed as more environment-friendly methods compared to traditional chemical approaches. Palmitoleic acid has been reported to be an effective algicidal bioactive compound against HABs, which were isolated from the bacterium [7]. In previous studies, PA exposure induced a series of immune, antioxidant and oxidation-related responses (e.g., SOD, ACP, ALP, LDH, and lysozyme activities, as well as MDA, GSH, NO, and total protein levels) in the hemolymph of bay scallops, and then caused oxygenic stresses [13,14], rendering them sensitive to PA exposure. Nevertheless, the molecular response of scallops to PA exposure is not well illuminated. On the basis of our previous works, these results of this transcriptome information could supplement the descriptions of toxicity of PA exposure at 80 mg/L, and then provide directions and insights for future investigations referring to pollutant toxicity models in scallops. The RNA-seq expression values and qPCR fold-change values were calculated on the basis of RPKM [21] and the MNE method and incorporated reference gene, respectively [22]. For transcripts quantified by using both methods, the directions of change in the present investigation were always the same and the magnitude of the fold-change in abundance was very similar. In addition, the current study is also the first report which is related to transcriptomic responses of scallop following PA exposure using deep-sequencing technology. Moreover, we observed that the quantitative values of some transcripts were significantly increasing such as “Unigene44066_All” (log2. Fold Change = 11.695), “Unigene31467_All” (log2. Fold Change = 11.689) and CL3015.Contig1_All (log2. Fold Change = 10.926) (Supplementary file 1) without functional annotation. Although their specific function remains unclear, the substantial up regulation found in the present work might be an indicative of relevant role during environmental stress responses. Therefore, we speculate that these genes may be involved in eliminating xenobiotics or apoptosis which was induced by PA exposure.

The biotransformation process is divided into two categories: phase I and phase II reactions [1]. Phase II reactions are catalyzed by GST to catalyzes the conjugation of reduced glutathione to reactive electrophiles of endogenous and exogenous compounds. Hence, GST activity is widely selected as a biomarker of contaminants in bivalves [1,23]. In the current study, variation in *GST* mRNA expression, including that of *GST1*, *GST2*, *GST3*, *GST-ω*, and *GST-A* was induced in the gills of PA-exposed bay scallops compared with the control group in the DGE library. These results imply that *GST* expression could be induced by PA exposure. This finding helps us to comprehend the molecular mechanism of the GSH increase observed in our previous work, which showed that GSH levels in the bay scallop exposed to 80 mg/L PA increased significantly at 48 h post-exposure [14], and subsequently stimulated the antioxidant or detoxification capacity of the PA-exposed scallops. Another important gene involving to the antioxidant defense system is SOD, which eliminates the ROS that can cause DNA damage or lipid peroxidation [24]. Our present results showing that the expression levels of *Cu/Zn SOD* and *Mn SOD* were up-regulated in the gills of bay scallop following to 80 mg/L PA exposure up to 48 h. These results are consistent with the findings of our previous study, in which we found that the expression levels of both *Mn SOD* and *Cu/Zn SOD* in the hemolymph of PA-exposed bay scallop increased at 48 h post-exposure [14]. These results indicate that PA exposure can induce *Cu/Zn SOD* and *Mn SOD* mRNA expression in both the gill and hemolymph when scallops are exposed to PA. Thus, expression of *Cu/Zn SOD* and *Mn SOD* in the gills of scallops could reflect PA exposure stress at high concentrations and provoke antioxidant defenses in scallops.

NAD(P)H:quinone reductase (NQR), known Martas DT-diaphorase, catalyzes the two-electron reduction of quinones and quinoid compounds [25]. This enzyme protects cells from redox cycling and oxidative stress [26]. *NQR* gene expression can be induced by antioxidants, oxidants, xenobiotics, heavy metals, and ionizing radiations, and is a part of a cellular defense mechanism that is responsible for the induction of more than two dozen genes in response to electrophilic and/or oxidative stress generated due to chemical exposure [26]. In the current work, we clearly observed that the expression of *NQR2* significantly declined after exposure to PA, indicating that PA might be an inhibitor of *NQR2* and suppress its expression. Therefore, it can be deduced that PA exposure may cause the diminishing of the protective ability against the toxicity of quinone-structured compounds. Genes encoding detoxification enzymes play irreplaceable roles in bivalves during metabolized foreign compounds or xenobiotics exposure such as toxicants, drugs, and chemical contaminants [3]. Among the DEGs detected in the current work, we identified several genes related to detoxification. The cytochrome P450 family is a major family of enzymes involved in the primary or phase I metabolism of xenobiotics such as drugs, pesticides, fatty acid, and toxins [27]. It was found that PA induced the expression of *CYP2C14*, *CYP2U1*, *CYP3A24*, and *CYP4A2*, suggesting they are crucial in metabolism and clearance of PA, and may facilitate the biotransformation of PA and accelerate its excretion, and could be regarded a good candidate biomarker for monitoring stress associated with PA exposure. However, the expression of *CYP4F22*, *CYP4B1*, and *CYP2C8* displayed an opposite response. This could suggest that PA exposure inhibits cytochrome P450 enzymes via genes expression of *CYP4F22*, *CYP4B1*, and *CYP2C8*, which may hinder or disturb the biotransformation of various other endogenous or xenobiotics in the bay scallop and even result in their accumulation. ATP-binding cassette (ABC) transporters belong to transmembrane proteins. They are responsible for transporting various structurally diverse substrates across biological membranes depending on ATP [28]. In addition, in aquatic organisms, they are responsible for multixenobiotic resistance phenotype by exporting xenobiotics out of the cells or by facilitating the sequestration of toxins within specialized cells or organelles, effectively segregating them away from vulnerable protein and DNA targets [28]. In our current results, it was found that the *ABCB1*, *ABCC1*, and *ABCG2* were down-regulated in bay scallops following PA exposure at 80 mg/L. These findings are in keeping with the previous research reporting that ABC transporters in mussel were up-regulated after exposure to *Prorocentrum lima* which produces okadaic acid. Huang et al. [29] also reported that the relative expression of P-glycoprotein gene, which belongs to the family of ATP-binding cassette (ABC) transporters, in the gills of *Perna viridis* increased significantly after exposure to *Perna lima*. These results indicate that PA could inhibit the *ABCB1*, *ABCC1*, and *ABCG2* expression, and then suppress exogenous resistance ability. ACP is a kind of important hydrolytic enzyme in phagocytic lysosomes [30]. In this work, it was found that the expression of *ACP* mRNA decreased in the gills of bay scallops after PA exposure, and then making a dent in the elimination of pathogens or phagocytized microorganisms in the PA-exposed gill.

The cathepsin proteins are consisted of lysosomal proteolytic enzymes that act a vital role in maintaining homeostasis in species. Particularly, these enzymes are in connection with intracellular protein turn over, degradation, immune response, and antigen processing [31,32]. Cathepsin B has been reported for its role in proteins degradation related to the lysosomal system associated with a variety of physiological and pathological processes such as infection, clearance, inflammation, and apoptosis [31]. Cathepsin B may induce or activate apoptotic pathways, once it is released from the lysosomes into the cytoplasm [33]. In previous research, it was reported that the relative expression level of the cathespin B mRNA was significantly increasing in the mantle and liver tissues of clams after being challenged with *Vibrio anguillarum* compared with control, which indicates that cathepsin B is an inducible acute phase and constitutively active protein [34]. It was also found that cathepsin B was potentially inducible in rock bream after exposure to *Edwardsiella tarda* and lipopolysaccharide, which indicates that it may act a role in the immune system [31]. Moreover, cathepsin B was also enhanced in the olive flounder following immersion in a suspension of *Flavobacterium columnare*, which suggested that cathepsin B regulation is an indispensable component of the immune responses during bacterial infections. In this present study, the expression level of *CTS-B* (cathepsin B) in bay scallop was found to be down-regulated after exposure to PA up to 48 h, suggesting that although PA exposure may not induce the lysosomal apoptosis pathway via *CTS-B*. However, the expression of *CTS-A* was stimulated during PA exposure, which it is speculated that PA exposure may impact on intracellular protein degradation/turn over, antigen processing, and immune responses by regulating *CTS-A*. 

## 4. Materials and Methods

### 4.1. Animal Maintenance and Palmitoleic Acid Exposure

All of bay scallops, weighing 48.02 ± 2.81 g, averaging length 6–7 cm, were obtained from the Noryangjin fisheries wholesale market, Seoul, South Korea. Scallops were acclimatized to laboratory conditions for 2 weeks before processing, maintained in lantern nets suspended in 800-L tanks with filtered and aerated sea water (temperature: 10 ± 1 °C; salinity: 30 ± 0.1‰). They were fed with Instant Algae^®^ Shellfish Diet (Reed Mariculture Inc., Campbell, CA, USA) at a rate of approximately 1.2 × 10^10^ algal cells/scallop/day. Half of the seawater was changed daily. 

For palmitoleic acid (PA) exposure, the bay scallops were divided into two groups: (1) control; (2) PA exposed group. Analytical grade PA (Sigma–Aldrich, USA) was dissolved in dimethyl sulphoxide (DMSO) [13]. The scallops in the PA-treated group were exposed to 80 mg/L PA. The control was exposed with an equal volume of DMSO. The final DMSO concentration was 0.0125‱ in all aquaria. Following PA exposure up to 48 h, gills were sampled from 18 scallops (i.e., six scallops with three replicates), stored in 1 mL TRIzol reagent (Invitrogen, USA) at −80 °C until use. Samples from 6 scallops were pooled for each replicate for RNA extraction.

### 4.2. RNA Preparation

Total RNA was extracted following the manufacturer’s instruction of TRIzol (Invitrogen, USA). The RNA contamination and degradation was accessed by agarose gels (1%) electrophoresis. The RNA purity and contamination was measured using a NanoPhotometer^®^ spectrophotometer (IMPLEN, CA, USA) and a Qubit^®^ RNA Assay Kit and a Qubit^®^ 2.0 Flurometer (Life Technologies, CA, USA) respectively. RNA integrity was accessed using the RNA Nano 6000 Assay Kit of the Agilent Bioanalyzer 2100 system (Agilent Technologies, CA, USA) [35].

### 4.3. Library Preparation and Illumina Sequencing

Here, 200 ng of DNase I-treated total RNA were purified by using oligo-dT beads. Fragment Buffer was used to cleave the polyA-containing mRNA into short fragments. During the RNA purification, ribosomal and other non-messenger RNA were also removed. First-strand cDNA was synthesized by reverse transcription using the mRNA fragments as the templates with adding First Strand Master Mix (Illumina, USA) and Super Script II (Invitrogen, USA). The second-strand cDNA was synthesized using Second Strand Master Mix (Illumina, USA). Then, the overhangs resulting from fragmentation were converted into blunt ends by using an End Repair Mix. After Adenylate 3’Ends DNA, the ligation reaction was performed by using RNA Index Adapter and Ligation Mix. Next, PCR Primer Cocktail and PCR Master Mix were added to enrich the cDNA fragments. Afterwards, cDNA fragments (260 bp in length) were selected for PCR amplification. The final library was quantified by qPCR with loading 1 μL of resuspended construct on an Agilent Technologies 2100 Bioanalyzer using Agilent DNA 1000. For cluster generation, the qualified and quantified libraries were first amplified within the flow cell on the cBot instrument (HiSeq^®^ 4000 PE Cluster Kit, Illumina). For paired-end sequencing, the clustered flow cell was then loaded onto the HiSeq 4000 Sequencer (HiSeq^®^ 4000 SBS Kit, Illumina) with the recommended read length of 100 bp. Both the library preparation and Illumina sequencing were conducted by Beijing Genomics Institute (BGI) (Hongkong, China).

### 4.4. De Novo Transcriptome Assembly

All the raw reads (reads with adaptors, low-quality reads, and reads in which unknown bases (N) comprised more than 5% of the read) were removed to generate clean data using SOAPnuke software. Clean reads were stored as the FASTQ format file [36], and then were assembled using the Trinity software to obtain unigenes. The resulting sequences assembled using Trinity was referred to as transcripts. Then, gene family clustering was performed using TGICL (TIGR Gene Indices clustering tools) to acquire the final unigenes. The unigenes were classified to two categories: (1) clusters, labeled by the prefix ‘CL’, followed by the cluster ID; (2) singletons presented by the prefix ‘unigene’. 

### 4.5. Gene Annotation and Analysis

The following seven databases were used functional annotation: the non-redundant protein sequence database (Nr), NCBI nucleotide database (Nt), Gene Ontology (GO), the Clusters of Orthologous Groups (COG), the Kyoto Encyclopedia of Genes and Genomes (KEGG), Swiss-Prot, and the Interpro database for unigenes. The unigenes were aligned to Nt, NR, COG, KEGG and SwissProt using Blast (version: v2.2.23) to obtain annotations [37]. To obtain GO annotations in conjunction with Nr annotations and the InterPro annotations, Blast2GO (version: v2.5.0) and InterProScan5 (version: v5.11-51.0) were used, respectively [38].

### 4.6. Enrichment Analysis of Differentially Expressed Genes

In order to map the high-quality reads to the reference unigene sequences, Bowtie (version: 2.2.5) was applied [39]. The unigene expression level calculation was performed by using RSEM (version: v1.2.12) [40]. We detected DEGs with PossionDis as requested. PossionDis is based on the Poisson distribution, performed as described by Audic and Claverie [41]. Furthermore, the thresholds were FDR (false discovery rate) <0.001 and fold change ≥2.00 to determine the significance of the differential gene expression.

The DEGs were classified according to the standard classification based on GO annotation results. GO and pathway functional enrichment analysis were also processed using phyper, a function of R. The formula for *p* value calculation in the hypergeometric test is as follows:

$$P=1-\overset{m-1}{\underset{i=0}{\sum }}\frac{\left(\begin{array}{c} M\\ i \end{array}\right)\left(\begin{array}{c} N-M\\ n-i \end{array}\right)}{\left(\begin{array}{c} N\\ n \end{array}\right)}\;$$

FDR was used for correction of each *p* value. A FDR < 0.001 was identified as significant enrichment.

### 4.7. Quantitative Real-Time PCR Verification

The expression profile of nine genes, which are related to toxification, antioxidant ability, and immunology, were performed using the qPCR technique. In a preliminary study, efficacy of various reference genes was evaluated following 80 mg/L PA exposure to scallop up to 48 h. A Web-based comprehensive tool RefFinder (https://omictools.com/reffinder-tool) was used to compare and rank candidate reference genes based on the rankings from three algorithms: GeNorm, NormFinder, and BeestKeeper. Thus, *β-actin* was selected as the housekeeping gene in this study. All specific primers used for qPCR are listed in Table 5. The first-strand cDNA was synthesized with 500 ng of total RNA using a PrimeScriptTM RT Reagent Kit (TaKaRa Bio, Japan). The qPCR amplifications were processed by using QiagenRotor-Gene Q RT-PCR Detection System (Qiagen, Hilden, Germany) in 12-μL reactions containing the following components: 1 μL forward primers (10 μM), 1 μL reverse primers (10 μM), 1 μL cDNA (50 ng), and 6.25 μL SYBR Premix Ex TaqTM TaKaRa Bio, Japan), and 2.75 μL ultra-pure water. The reaction profile was as following: one cycle of 2 min at 94 °C; 40 cycles of 94 °C for 20 s, 30 s at 58 °C, and 40 s at 72 °C for extension [42]. Melting curve analysis was performed following amplifications The amplification efficiencies were between 90% and 110%, and the correlation coefficients (R2) of all standard curves were >0.99. The relative expression ratios of the target genes were calculated with the method described by Livak K.J. [43]. In all cases, Ct values were determined based on three biological replicates each with two technical replicates. 

All data was analyzed using statistical software SPSS 19.0 (IBM Corp., Armonk, NY, USA). The differences were tested by LSD test. *p*-values <0.05 indicates a statistical significance. The values were presented as mean ± standard deviation (SD) of the gene expression.

## 5. Conclusions

In conclusion, we present herein an extensive investigation of the PA-sensitive genes with differential expressions in gills of bay scallops. These genes are involved in a serial of immune and detoxification processes against to PA. The current results not only demonstrate the transcriptional complexity of the immune response to PA in scallops, but also suggest the possibility of identifying the genes implicated in regulating bivalve tolerance to xenobiotic stress. Illumina next-generation sequencing technology provides abundant resources for investigating the molecular basis underlying the effects of PA in scallops and for understanding the immune- and detoxification-associated mechanisms that counteract the harmful effect of this compound in these organisms. Moreover, it is also supplements and reinforcing findings of our previous investigation, in which we established a strong cause and effect relationship between PA and physiological responses in bay scallops. PA might act as an environmental endocrine disruptor, which could negatively affect the endocrine system, or affect the signal transduction pathway in scallops. Therefore, this study further verifies a potential risk of popularizing and applying of PA as an algaecide management in scallop production. However, shorter exposure time and lower concentration of PA may have less adverse effects on scallops. In addition, the use of suitable immunostimulants synergistically with PA in scallop culture might be an effective alternative method to boost scallop immune system function as well as reduce the negative effect of PA. Furthermore, the results of this study highlight the need to arouse researchers’ attention to investigate the impacts of using some other algaecides to control the outbreak of algal blooms in the marine environment, as well as address secondary pollution caused by PA.

## Figures and Tables

**Table 1:** Summary of sequencing reads after filtering.

Sample	Total Raw Reads (Mb)	Total Clean Reads (Mb)	Total Clean Bases (Gb)	Clean Reads Q20 (%)	Clean Reads Q30 (%)	Clean Reads Ratio (%)
CN	45.92	45.76	4.58	98.21	95.30	99.65
PA	45.92	45.76	4.58	98.40	95.69	99.65

PA: palmitoleic acid; CN: control.

**Table 2:** Quality metrics of transcripts.

Sample	Total Number	Total Length	Mean Length	N50	N70	N90	GC (%)
CN	78,434	52,871,582	674	1230	568	252	39.13
PA	80,539	56,824,660	705	1308	610	261	39.60

GC (%): the percentage of G and C bases in all unigenes.

**Table 3:** Quality metrics of unigenes.

Sample	Total Number	Total Length	Mean Length	N50	N70	N90	GC (%)
CN	51,495	41,085,320	797	1412	702	302	39.47
PA	55,973	46,742,302	835	1503	753	313	39.86
All unigenes	62,099	57,266,803	922	1735	897	338	39.51

N50: a weighted median statistic that 50% of the total length is contained in unigenes great than or equal to this value. GC (%): the percentage of G and C bases in all unigenes.

**Table 4:** Detoxification and immune-related differentially expressed genes in gills of bay scallop exposed to 80 mg/L PA up to 48 h.

Description	Transcript	Log2 Fold Change (RNA-seq)	Regulation
**Down regulated**			
Immune system	*CLEC4* *E*	−4.278	Down
	*CLEC4* *F*	−9.069	Down
	*FIBCD 1*	−2.224	Down
	*ACP*	−1.088	Down
Apoptosis	*CTS-B*	−1.25	Down
	*BIRC7*	−14.271	Down
Transmembrane proteins	*ABCB1*	−1.481	Down
	*ABCC1*	−1.167	Down
	*ABCG2*	−1.514	Down
Oxidative stress	*NQR2*	−8.406	Down
Metabolism of xenobiotics	*CYP 4F22*	−7.988	Down
	*CYP 4B1*	−8.778	Down
	*CYP 2C8*	−3.918	Down
	*CREB 3*	−1.147	Down
Antioxidant	*GR*	−5.628	Down
	*Nrf2*	−1.035	Down
**Up regulated**			
Antioxidant	*Cu/Zn SOD*	2.107	Up
	*Mn SOD*	1.728	Up
	*GST 1*	2.860	Up
	*GST 2*	10.276	Up
	*GST 3*	3.237	Up
	*GST ω*	1.198	Up
	*GST A*	2.648	Up
	*GST κ*	5.319	Up
Apoptosis	*CTS-A*	1.07	Up
Immune system	*TLR 2*	1.780	Up
	*TLR 4*	4.268	Up
	*TLR 13*	5.349	Up
Metabolism of xenobiotics	*CYP 2C14*	8.683	Up
	*CYP 2U1*	4.241	Up
	*CYP 3A24*	1.219	Up
	*CYP 4A2*	1.786	Up
	*mo-A*	1.86	Up

CLEC4 = C-type lectin domain family 4; FIBCD = fibrinogen C domain-containing protein; TLR = toll-like receptor; ACP = acid phosphatase; ABC = ATP-binding cassette sub-family; GST = glutathione *S*-transferase; GR = glutathione reductase; CREB = cyclic AMP-responsive element-binding protein; Cu/Zn SOD = copper/zinc superoxide dismutase; Mn SOD = manganese superoxide dismutase; CYP = cytochrome P450; Nrf2 = nuclear factor erythroid 2-related factor 2; CTS-A = cathepsin A; CTS-B = cathepsin B; mo-A = monoamine oxidase A; NQR2 = NADPH2:quinone reductase; BIRC7 = baculoviral IAP repeat-containing protein 7.

**Table 5:** All primers used in our validation analysis.

Primer		Products
Primers Used for *Fe/Mn SOD*		
*Mn SOD*-F	5’GTGCCTCTACTGCTGTCC3’	103 bp
*Mn SOD*-R	5’TGAAGTGGGTCCTGGTTA3’	
Primers used for *GST-Omega*		
*GST ω*-F	5’GGCAAACCCGCTTCTGTA3’	213 bp
*GST ω*-R	5’CCCGTGCTGTGGGATAAA3’	
Primers used for *GST-Kappa*		
*GST κ*-F	5’GACCAAGGGATTTCTGATGAG3’	183 bp
*GST κ*-R	5’ATGAGCGACAATAATAGGGGAT3’	
Primers used for *ACP*		
*ACP*-F	5’AGACAGAACCCGACAACTC3’	266 bp
*ACP*-R	5’GCTATGAGGCTGATTAGAAGG3’	
Primers used for *CTS-A*		
*CTS-A-F*	5’ACCGCCCTTGACAGTAAC3’	203 bp
*CTS-A-R*	5’CACGAAAGAAACGAGTAA3’	
Primers used for *CTS-B*		
*CTS-B*-F	5’ACCCTCCGTCACATCCCA3’	205 bp
*CTS-B*-R	5’GACTCCCGTAAGGCGTGGT3’	
Primers used for mo-A		
*mo-A*-F	5’GGGGAGCATCTTTAATCG3’	136 bp
*mo-A*-R	5’ACTGGCTGGTTCTTCACA3’	
Primers used for NQR2		
*NQR2-A*-F	5’CCAAGTACAACTACGGCTCT3’	270 bp
*NQR2-A*-R	5’AAGGTCGGGAAACTATGC3’	
Primers used for *CYP3A24*		
*CYP3A24*-F	5’AGCAGCCAATGAGGAGTGT3’	198 bp
*CYP3A24*-R	5’TGTAGGACCATATCGCAGAC3’	

## Supplementary Materials

The following are available online at http://www.mdpi.com/2218-273X/8/4/139/s1. Supplementary file 1: The differential expression unigenes between the control and the PA-treated groups. The accession number for our Raw dataset was submitted to GEO database under the accession number GSE121110.
